# APOE ε4 and amyloid status moderate the associations between sleep, physical activity, and tau‐PET burden in cognitively unimpaired older adults

**DOI:** 10.1002/alz.71555

**Published:** 2026-06-16

**Authors:** Daniel D. Callow, Nisha Rani, J. Carson Smith, Anja Soldan, Corinne Pettigrew, Adam P. Spira, Marilyn Albert, Arnold Bakker, BIOCARD Research Team

**Affiliations:** ^1^ Department of Psychiatry and Behavioral Sciences Johns Hopkins University School of Medicine Baltimore Maryland USA; ^2^ Department of Kinesiology University of Maryland College Park Maryland USA; ^3^ Department of Neurology Johns Hopkins University School of Medicine Baltimore Maryland USA; ^4^ Department of Mental Health Johns Hopkins Bloomberg School of Public Health Baltimore Maryland USA; ^5^ Johns Hopkins Center on Aging and Health Baltimore Maryland USA

**Keywords:** actigraphy, exercise, genetic risk, preclinical Alzheimer's disease, sleep, tau positron emission tomography (PET)

## Abstract

**INTRODUCTION:**

Disturbed sleep and physical inactivity are associated with increased risk for dementia. However, associations with Alzheimer's disease (AD) pathology, and whether these associations differ depending on underlying disease risk, remain unclear.

**METHODS:**

We examined associations of actigraphy‐derived total volume of physical activity (TVPA), total sleep time (TST), and wake after sleep onset (WASO) with tau burden measured by positron emission tomography (PET) in Braak I–II subregions among 120 cognitively unimpaired older adults. Moderation by *APOE* ε4 carrier status and amyloid positivity, based on amyloid PET, was examined to evaluate risk‐dependent associations.

**RESULTS:**

Greater WASO was associated with higher tau burden among *APOE* ε4 carriers and amyloid beta (Aβ)‐positive individuals. TVPA was associated with higher tau burden among Aβ‐positive individuals and lower tau burden among Aβ‐negative individuals.

**DISCUSSION:**

Associations of sleep and physical activity with early tau pathology differ by genetic risk and Aβ status, highlighting the importance of risk stratification.

## Highlights

- Actigraphy‐measured sleep and activity were examined in relation to early tau PET.
- Greater wake after sleep onset related to higher Braak I–II tau in at‐risk adults.
- Physical activity–tau associations differed by amyloid status.
- No associations were observed without genetic or amyloid risk stratification.
- Findings show risk‐dependent heterogeneity in lifestyle–tau relationships.

## 1 BACKGROUND

Evidence suggests that modification of certain lifestyle factors may substantially reduce the risk of dementia.^1^, ^2^ Disrupted sleep and reduced physical activity are two widely studied lifestyle factors and have been consistently associated with increased risk of cognitive decline and incident dementia.^3^, ^4^ However, the extent to which these behaviors are associated with in vivo markers of Alzheimer's disease (AD) pathology, particularly in cognitively unimpaired individuals, remains unclear. Moreover, accumulating evidence suggests that associations between lifestyle behaviors and AD biomarkers may differ depending on underlying disease risk, including genetic susceptibility and amyloid burden.^5^, ^6^, ^7^, ^8^

Experimental studies in animal models demonstrate that sleep disruption is associated with increased amyloid beta (Aβ) and tau accumulation.^9^, ^10^ In humans, poorer sleep quality and shorter sleep duration have been linked to greater AD pathological burden across the AD spectrum, most consistently using cerebrospinal fluid (CSF) biomarkers and amyloid positron emission tomography (PET) imaging.^11^, ^12^, ^13^, ^14^, ^15^, ^16^ These associations appear to be stronger, or detectable primarily, among individuals at elevated genetic risk for AD, such as carriers of the *ε4 allele of the apolipoprotein E gene (APOE ε4)*.^5^, ^17^, ^18^, ^19^ Objective actigraphy‐derived sleep measures, including total sleep time (TST) and wake after sleep onset (WASO), provide reliable assessments of habitual sleep patterns and have been linked to amyloid burden in cognitively unimpaired samples.^14^, ^15^ However, evidence linking objectively measured sleep to tau pathology assessed using tau PET imaging remains limited, particularly among cognitively unimpaired individuals during the earliest stages of tau accumulation.

Evidence linking physical activity to AD pathology in humans has focused largely on amyloid biomarkers and self‐reported physical activity measures. Several reviews of this literature highlight that associations between physical activity and amyloid burden are generally modest and inconsistent, varying by disease stage, genetic risk, and physical activity and amyloid measurement modality.^20^, ^21^, ^22^ Additional studies further suggest that associations may be most evident among cognitively unimpaired individuals at elevated AD risk, including *APOE* ε4 carriers, whereas other investigations report null findings.^7^, ^23^, ^24^ Moreover, substantially less is known about the relationship between objectively measured physical activity and tau pathology assessed using tau‐PET imaging. The few existing studies have largely relied on self‐reported activity measures, fluid biomarkers such as phosphorylated tau, and diagnostically heterogeneous samples of older adults.^20^, ^24^, ^25^ With the exception of a recent longitudinal study reporting slower neocortical tau accumulation among physically active, Aβ‐positive‐positive individuals,^8^ evidence linking physical activity to tau‐PET burden, particularly within regions showing early accumulation of tau and among cognitively unimpaired adults, remains sparse.

Tau‐PET imaging is particularly valuable when examining these associations, as it can capture the spatial progression of tau pathology in vivo in the earliest stages of AD. Neuropathological studies demonstrate that tau pathology first emerges in transentorhinal and entorhinal regions before extending into hippocampal and neocortical areas, forming the basis of the Braak staging framework (I‐VI).^26^, ^27^, ^28^ Recent advances in tau‐PET quantification now enable sensitive detection of tau burden within Braak I^–^II subregions prior to symptoms of overt clinical impairment, and tau accumulation in these early Braak subregions are shown to predict subsequent cognitive decline during the preclinical phase of AD.^29^, ^30^, ^31^

In cognitively unimpaired older adults, in particular, associations of sleep and physical activity with AD‐related pathology may not be uniform.^32^ However, prior studies largely examined these relationships in aggregate samples without testing whether associations differed by biological risk (e.g., APOE ε4 status or amyloid burden), potentially obscuring meaningful heterogeneity. Emerging evidence suggests that associations may be strongest in at‐risk individuals,^8^, ^19^ but this has not been systematically examined using objective lifestyle measures and tau‐PET imaging in cognitively unimpaired populations.

To address these gaps, this study examined whether associations of actigraphy‐derived sleep and physical activity with early medial temporal lobe tau pathology differed by biological risk status in cognitively unimpaired older adults. Specifically, we tested moderation by APOE ε4 carrier status and Aβ positivity. We hypothesized that associations of physical activity and sleep with early tau accumulation would be stronger among ε4 carriers and individuals with elevated amyloid burden.

## 2 METHODS

### 2.1 Study design

This study used data from participants in the Biomarkers of Cognitive Decline among Normal Individuals (BIOCARD) study, a longitudinal cohort initiated in 1995 at the National Institutes of Health. The original cohort included 349 middle‐aged adults, approximately 75% of whom reported a family history of dementia. Participants underwent comprehensive clinical assessments, cognitive evaluations, CSF and blood collection, and 1.5T magnetic resonance imaging (MRI) scans. The study was stopped for administrative reasons in 2005 and was reinitiated at Johns Hopkins University (JHU) in 2009. Since then, participants have continued annual evaluations, including clinical assessments and cognitive testing. Neuroimaging, including 3T MRI, has been conducted biennially since 2015. The acquisition of Aβ‐positive scans began in 2015, with tau‐PET acquisition commencing in 2021. Additionally, the biennial collection of wrist actigraphy data, used to quantify physical activity and sleep, began in 2015 during alternating years when neuroimaging was not performed. A total of 151 new participants were enrolled at JHU from 2021 to July 2025 to increase the size and diversity of the cohort.

**RESEARCH IN CONTEXT**

1. **Systematic review**: We reviewed prior literature examining associations between sleep, physical activity, and AD biomarkers, including amyloid and tau, with an emphasis on objective behavioral measures and tau PET imaging. While disturbed sleep and lower physical activity are consistently associated with dementia risk and amyloid burden, evidence linking objectively measured sleep and physical activity to early tau pathology in cognitively unimpaired individuals remains limited and mixed. Few studies have examined whether these relationships vary by genetic or amyloid risk.
2. **Interpretation**: In cognitively unimpaired older adults, associations between sleep continuity, physical activity, and early tau PET burden differed by APOE ε4 carrier status and amyloid positivity. Sleep fragmentation was associated with higher tau burden only among higher‐risk individuals, and physical activity showed risk‐dependent associations by amyloid status. These findings suggest that risk stratification is critical for detecting relationships between modifiable behaviors and early tau pathology.
3. **Future directions**: Future intervention studies should test whether modifying sleep continuity or physical activity influences tau accumulation and should use genetic and amyloid risk stratification to identify individuals most likely to benefit.

This cross‐sectional analysis included all participants who were cognitively unimpaired at the time of the tau‐PET visit, had undergone both amyloid and tau‐PET imaging, and had usable actigraphy measurements relatively close in time to the PET scans (*N* = 120). Actigraphy data for the primary analyses were derived from each participant's first available actigraphy assessment, consistent with the study design emphasizing baseline lifestyle measures. The mean and median interval between actigraphy and tau‐PET imaging was −958 and −1121 days (standard deviation [SD] = 1110 days; range = −2579 to +656 days), with negative values indicating actigraphy assessments conducted prior to tau‐PET and positive values indicating assessments conducted afterward. All participants provided written informed consent, and study procedures were approved by the Institutional Review Board at JHU.

### 2.2 Clinical and cognitive assessments

Participants in the BIOCARD study undergo annual clinical and cognitive evaluations, including medical, psychiatric, and neurological assessments, a semi‐structured interview based on the Clinical Dementia Rating (CDR) scale,^33^ and a comprehensive neuropsychological battery.^34^ Consensus diagnoses are determined annually by the BIOCARD Clinical Core using procedures comparable to those of the National Institute on Aging Alzheimer's Disease Centers program, as described in prior publications.^34^ Syndromic diagnostic categories include cognitively unimpaired, mild cognitive impairment (MCI), impaired not MCI, and dementia.^35^ For the present study, analyses were restricted to participants classified as cognitively unimpaired at the time of tau‐PET imaging. Consistent with prior BIOCARD publications,^36^, ^37^ individuals classified as “impaired not MCI” were included in the cognitively unimpaired group.

### 2.3 Wrist‐worn actigraphy data collection

Participants were provided with an actigraph (Actiwatch‐2, Philips Respironics, Bend, OR, USA) to wear on their non‐dominant wrist for seven consecutive days, following the in‐person clinical/cognitive assessment. Participants were asked to complete daily logs documenting the removal of the actigraph, travel across time zones, naps, and sleep times. Throughout actigraphy monitoring, participants used the event marker to indicate the start of their intended sleep period at bedtime and to mark final awakening in the morning. Each morning, participants recorded in a sleep diary the times corresponding to intended sleep onset (“lights out”) and rising from bed for the day. Participants returned the actigraph via mail. The data were downloaded with Actiware Software (version 6.0.9, Philips Respironics) and processed without knowledge of the participants’ cognitive or clinical status. Data collected during participant‐reported travel across time zones, illness, periods of non‐wear, and any device malfunctions were deemed invalid and excluded from the analyses. Nights in which participants reported taking medications that could affect sleep patterns (e.g., trazodone, zolpidem) were labeled as non‐valid nights and excluded from subsequent analysis.

### 2.4 Quantification of sleep parameters

Two standard nighttime sleep parameters were extracted from valid nights using a widely used algorithm^38^: (1) TST (the number of minutes slept while in bed) and (2) WASO (total minutes awake between sleep onset and final awakening). These standard sleep parameters were averaged across valid nights and analyzed as continuous variables.

### 2.5 Quantification of physical activity

As in prior work,^36^, ^37^, ^39^ physical activity was objectively quantified with actigraphy accelerometer data collected in 30‐s epochs. Activity counts (validated measures of movement intensity) were calculated, with at least three full 24‐h periods of valid data required for inclusion.^40^−^42^ A valid day was defined as having fewer than 5% missing epochs. Missing data points were imputed using the participant's mean activity at the corresponding time of day from valid days. To normalize skewed distributions, we applied a log transformation to the activity counts: log(1 + counts per minute). These were aggregated across minutes and subsequently hours. Total volume of physical activity (TVPA) was computed by averaging activity counts during each day's 10 most active hours.^36^, ^37^, ^39^

### 2.6 Amyloid and tau PET acquisition

PET scans were acquired with a GE DISCOVERY RX PET/CT scanner. For tau‐PET imaging, images were acquired for 30 min approximately 90 min after participants were injected with ^18^F‐MK6240, a fluorine‐18–labeled tau PET radiotracer with high affinity for paired helical filament tau pathology associated with Alzheimer’s disease. For amyloid PET, images were acquired for 20 min approximately 50 min after participants were injected with ^11^C‐PiB, or carbon‐11–labeled Pittsburgh compound B, an amyloid PET radiotracer that binds fibrillar amyloid‐β plaques. Standard reconstruction techniques included 3D ordinary Poisson ordered‐subset expectation maximization, with corrections applied for detector efficiency, attenuation, scatter, and radioactive decay. Standardized uptake values were computed relative to participant weight and injected dose.

### 2.7 PET image analysis

PET data were smoothed using a 6‐mm Gaussian kernel and aligned using rigid‐body registration. Mean PET volumes were registered to each subject's high‐resolution T1‐weighted MRI using FreeSurfer. For partial volume correction, we used a region‐based, voxel‐wise method employing the geometric transfer matrix informed by tissue segmentation. For quantification, we calculated standardized uptake value ratios (SUVRs) using the pons (tau) and cerebellar gray matter (amyloid) as reference regions.^43^

### 2.8 Tau‐PET burden quantification

As in our prior work,^44^−^46^ tau burden was evaluated within anatomically defined regions of interest (ROIs) corresponding to Braak stages I and II, derived from high‐resolution medial temporal lobe segmentations using Advanced Segmentation of Hippocampal Subfields software tailored for older adults. Braak stage I included the entorhinal cortex and Brodmann area 35 (transentorhinal cortex), the earliest site of tau deposition. Stage II included Brodmann area 36 and anterior/posterior hippocampal regions. SUVR values were averaged across these ROIs to produce a composite measure of early tau accumulation.^47^, ^48^

### 2.9 Amyloid burden quantification

Global amyloid burden was quantified by averaging ^11^C‐PiB uptake across several amyloid‐associated regions, including the medial and lateral orbitofrontal cortex, precuneus, cingulate cortex, parietal cortex, temporal cortex, and superior frontal gyrus. A mean SUVR threshold of 1.40 was used to define amyloid positivity, consistent with prior studies.^49^ Amyloid‐ and tau‐PET scans were typically acquired at the same visit; across the full sample, the mean interval between scans was 110 days (median = 1 day).

### 2.10 APOE genotype

Genotyping of the *APOE* gene was performed via restriction enzyme digestion of PCR‐amplified genomic DNA. Participants were classified as ε4 carriers if they had at least one copy of the ε4 allele; all others were categorized as non‐carriers.^50^

### 2.11 Vascular risk factors

A composite vascular risk score was derived from participants’ medical history. This score, which was validated in prior studies,^51^ represents the total number of vascular risk factors present, with each factor coded as 1 (present, either recent or historical) or 0 (absent). The five components included hypertension, hypercholesterolemia, current smoking (within the past 30 days), obesity (body mass index ≥ 30 kg/m^2^), and diabetes. Data for these risk factors were collected at the same visit as the tau‐PET scan. The resulting vascular risk score was included as a covariate in regression models, as vascular health may confound the relationships between physical activity, sleep, and brain outcomes.

### 2.12 Statistical analysis

Differences between Aβ‐positive and Aβ‐negative as well as by *APOE* ε4 status were assessed by Wilcoxon signed‐rank tests for continuous variables and chi‐squared tests for categorical variables (Table S1 and S2). We used bivariate Pearson correlations to examine simple associations between the objectively measured physical activity and sleep parameter variables, controlling for age. Continuous actigraphy predictors were standardized prior to analysis.

Primary analyses were conducted using multivariable linear regression models. Mean tau‐PET burden in early Braak regions (i.e., average of SUVR in Braak stages I and II) was modeled as a function of TVPA, TST, and WASO, which were entered simultaneously as predictors. To evaluate potential moderation by biological risk, separate models were specified to test two‐way interactions between each lifestyle predictor (TVPA, TST, WASO) and APOE ε4 carrier status or Aβ‐positive status. In all interaction models, mean Braak I–II tau‐PET SUVR was specified as the outcome variable. Predictors included the focal lifestyle variable, the moderator (APOE ε4 status or Aβ status), their interaction term, and the main effects of all lifestyle variables (TVPA, TST, WASO) and covariates. All models included TVPA, TST, and WASO simultaneously to estimate the unique association of each lifestyle factor with tau‐PET burden while accounting for shared variance among these correlated behavioral measures. This approach allows for the interpretation of each lifestyle variable independent of the others and reduces potential confounding due to overlap between physical activity and sleep characteristics. All models controlled for age, sex, years of education, APOE ε4 carrier status, Aβ‐positive positivity, vascular risk score, actigraphy monitoring days, and the number of days between the actigraphy assessment and tau‐PET imaging. When significant interactions were identified, post hoc simple slopes analyses were conducted using estimated marginal trends to characterize the association between lifestyle predictors and tau burden within each subgroup (e.g., APOE ε4 carriers vs non‐carriers, Aβ‐positive vs Aβ‐negative individuals).

All multiple linear regression models were examined for influential data points such as abnormal leverage (hat leverage values > 3 times average), influence (Cook's D > 0.5), and discrepancy (studentized residuals > 3). Furthermore, collinearity between covariates and predictors in all models was checked for a high variance inflation factor (VIF > 5). No additional data were removed from further analyses based on an exclusionary criterion of violating more than one of these three heuristics. To account for multiple testing across the six interaction terms, we applied Benjamini‐Hochberg false discovery rate (FDR) and interpreted only those interaction effects with *p* values surviving correction.

We also conducted sensitivity analyses to determine whether the primary results for these models remained the same (a) after excluding participants with a diagnosis of “impaired not MCI” and (b) with amyloid burden modeled as a continuous variable instead of categorical as positive or negative. Finally, based on previous reports of a U‐shaped relationship between sleep duration and cognition, we conducted an additional sensitivity analysis with TST as a tertile categorical predictor for TST < 6 h, 6 to 8  (reference), and >8 h.^36^ In addition, to address potential temporal misalignment between lifestyle and imaging measures, we repeated all models using actigraphy data from the visit closest to tau‐PET and restricting the sample to participants with ≤750 days between assessments.

## 3 RESULTS

### 3.1 Participant demographics

Participant characteristics for the full sample (*N* = 120) are presented in Table 1, with additional participant characteristics broken down by Aβ‐positive and *APOE* ε4 carrier status in Table S1 and S2. Participants had a mean age of 69.5 years (SD = 9.3), and 59% were female. Thirty‐three individuals (28%) were classified as Aβ‐positive, while 43 (36%) were *APOE* ε4 carriers, and 18 (15%) were both Aβ‐positive and *APOE* ε4 carriers.

**TABLE 1:** Participant characteristics.

Participant characteristic	Full sample (*n* = 120)
Female sex, *n* (%)	71 (59%)
Age	69.5 ± 9.3
Education, years	17.5 ± 2.0
*APOE* ε4 carrier, *n* (%)	43 (36%)
*Amyloid PET positive, n (%)*	33 (28%)
TVPA^a^	2859 ± 325
Total sleep time (min)	459.4 ± 55
Wake after sleep onset (min)	40.8 ± 20.5
Tau‐PET SUVR in Braak I and II^b^	1.9 ± 0.7
Time from actigraphy to tau‐PET (days)	−958 ± 1,121
Vascular risk score	1.2 ± 0.9
Race / ethnicity, *n* (%)	
White, non‐Hispanic	110 (92%)
Black, non‐Hispanic	4 (3.3%)
Hispanic	5 (4.2%)
Other	1 (0.8%)

*Note*: Values are mean ± standard deviation unless otherwise indicated.

Abbreviations: PET = positron emission tomography; SD = standard deviation; SUVR = standardized uptake value ratio; TVPA = total volume of physical activity.

^a^ TVPA represents the average log‐transformed total activity epoch counts from the 10 most active hours per day.

^b^ Tau‐PET SUVR values reflect the mean uptake across Braak stages I and II.

### 3.2 Correlations between actigraphic physical activity and sleep measures

Across the full sample, greater TVPA was modestly associated with greater WASO (*r* = 0.20, *p *= 0.025), indicating that higher daytime activity levels were related to more fragmented sleep. TVPA was not associated with TST (*r* = −0.05, *p *= 0.607).

### 3.3 Independent associations of physical activity and sleep with early tau‐PET burden

In fully adjusted models, none of the actigraphic sleep or physical activity predictors were independently associated with early tau‐PET burden in Braak stages I‐II regions (all *p* ≥ 0.250). As expected, Aβ‐positive positivity was strongly associated with higher tau burden (*β* = 0.704, SE = 0.133, *p* < 0.001). Age showed a marginal, non‐significant positive association with tau‐PET burden (*β* = 0.013, SE = 0.008, *p* = 0.094), whereas sex, education, vascular risk, APOE ε4 status, actigraphy monitoring days, and the actigraphy‐to‐PET interval were not significantly related to tau burden (all *p* ≥ 0.110; Table 2).

**TABLE 2:** Associations between actigraphy‐derived physical activity, sleep measures, and tau‐PET burden in Braak I–II, and moderating effects of APOE ε4 status and amyloid positivity in cognitively unimpaired older adults.

Outcome	Predictor	Beta estimate	SE	*p* value
Main‐effects model				
Tau‐PET	TVPA	0.044	0.060	0.464
	TST	−0.025	0.059	0.668
	WASO	0.066	0.061	0.280
	APOE ε4 carrier status	0.199	0.123	0.110
	Aβ‐positive status	0.704	0.133	<0.001
	Age	0.013	0.008	0.095
	Sex	−0.141	0.117	0.233
	Education	0.010	0.029	0.715
	Time from actigraphy to PET	0.001	0.001	0.781
	Vascular risk score	0.019	0.068	0.784
	Actigraphy monitoring days	0.020	0.077	0.792
Separate models: APOE ε4 × lifestyle interactions				
Tau‐PET	TVPA × APOE ε4 carrier status	0.220	0.113	0.053
	TST × APOE ε4 carrier status	0.204	0.111	0.068
	WASO × APOE ε4 carrier status	0.319	0.109	0.004^*^
Separate models: Amyloid status × lifestyle interactions				
Tau‐PET	TVPA × Aβ‐positive status	0.347	0.109	0.002^*^
	TST × Aβ‐positive status	0.053	0.125	0.670
	WASO × Aβ‐positive status	0.384	0.112	<0.001^*^

*Note*: Linear regression models examined the independent associations of total volume of physical activity (TVPA), sleep duration (TST), and sleep quality (wake after sleep onset, WASO) with tau‐PET burden in Braak I–II regions. Each interaction term was tested in a separate regression model (i.e., interaction terms were not entered simultaneously). All models were adjusted for age, sex, years of education, APOE ε4 carrier status, Aβ‐positive positivity, vascular risk score, actigraphy monitoring days, and the number of days between the actigraphy assessment and tau‐PET imaging. Continuous actigraphy predictors were standardized prior to analysis.

* Significant after FDR correction for multiple comparisons.

### 3.4 Moderating effect of APOE ε4 status on actigraphy measures with early tau‐PET burden

**FIGURE 1:**
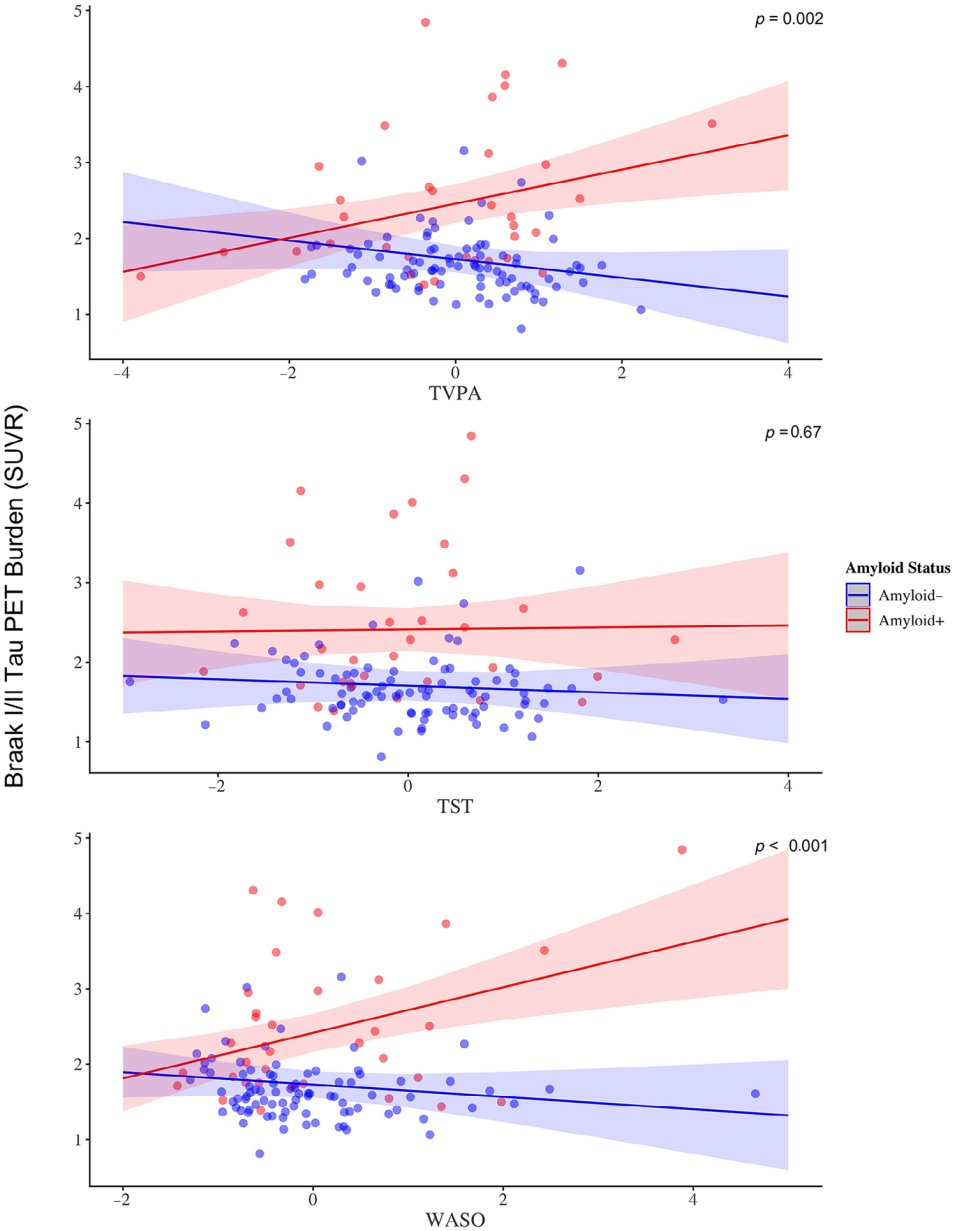
Moderating effect of APOE ε4 carrier status on associations between total volume of physical activity (TVPA), total sleep time (TST), and wake after sleep onset (WASO) and early tau‐PET burden. Only the WASO moderating effect survived multiple‐comparisons correction.

APOE ε4 carrier status significantly moderated the association between WASO and tau‐PET burden (*β* = 0.319, SE = 0.109, *p* = 0.004), which remained significant after FDR correction. Simple slope analyses indicated that greater WASO was associated with higher tau burden among APOE ε4 carriers (*β* = 0.238, SE = 0.083, 95% confidence interval [CI] [0.073, 0.403], *p* = 0.005), whereas no association was observed among non‐carriers (*β* = −0.081, SE = 0.077, 95% CI [−0.234, 0.072], *p* = 0.299). In contrast, interactions between APOE ε4 status and TVPA (*β* = 0.220, SE = 0.113, *p* = 0.053) and TST (*β* = 0.204, SE = 0.111, *p* = 0.068) did not survive correction for multiple comparisons. These results are shown in Figure 1 and presented in Table 2.

### 3.5 Moderating effect of Aβ‐positive status on actigraphy measures with early tau‐PET burden

**FIGURE 2:**
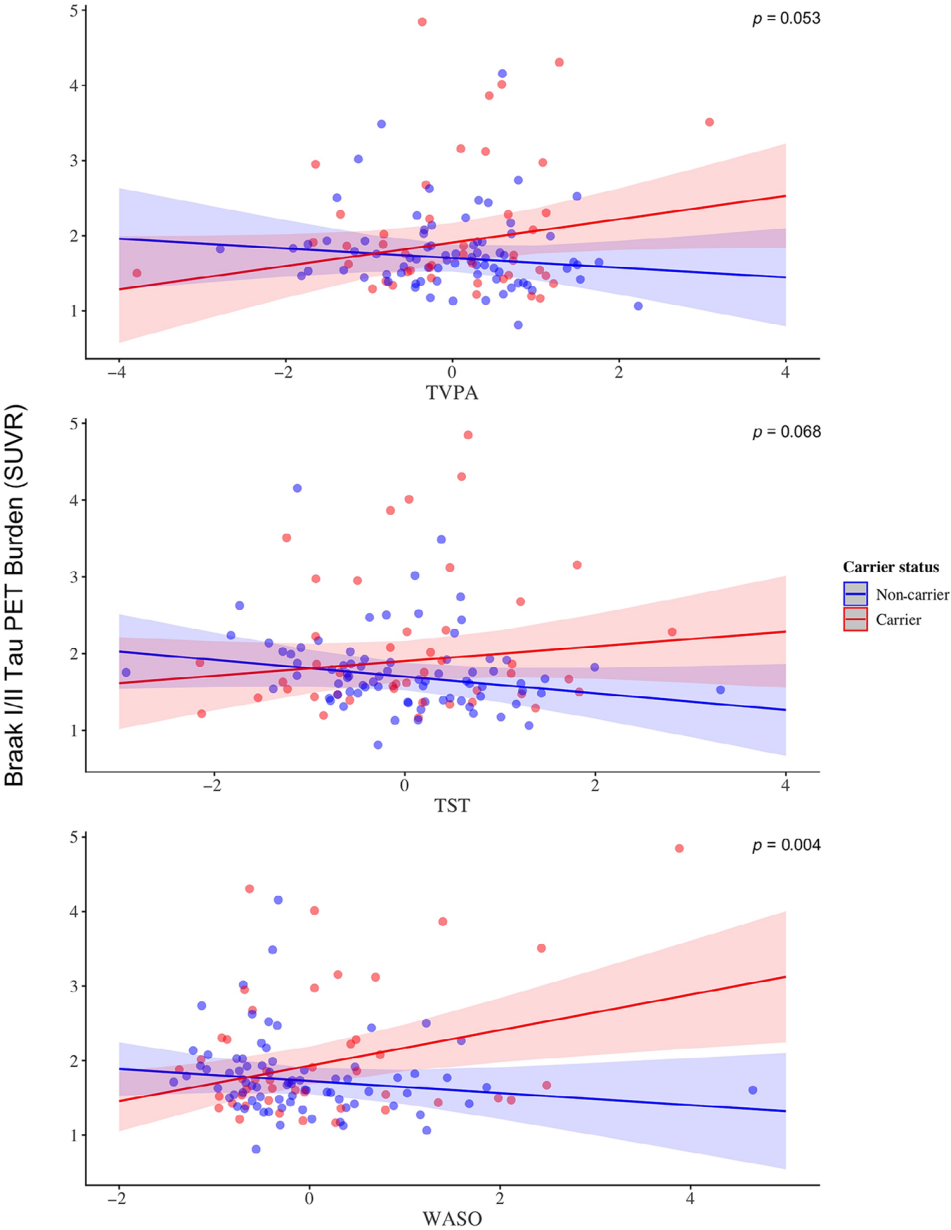
Moderating effect of Aβ‐positive positivity status on associations between total volume of physical activity (TVPA), total sleep time (TST), and wake after sleep onset (WASO) and early tau‐PET burden. Both the WASO and TVPA moderating effects survived multiple comparison correction.

Aβ‐positive status significantly moderated the associations of both total volume of physical activity (TVPA; *β* = 0.347, SE = 0.109, *p* = 0.002) and WASO (*β* = 0.384, SE = 0.112, *p* < 0.001) with Braak I‐II tau‐PET burden, and both interaction effects remained significant after FDR correction. In contrast, Aβ‐positive status did not significantly moderate the association between TST and tau‐PET burden (*β* = 0.053, SE = 0.125, *p* = 0.670). These results are depicted in Figure 2 and Table 2.

Simple slope analyses revealed that greater TVPA was associated with higher tau burden among Aβ‐positive individuals (*β* = 0.225, SE = 0.081, 95% CI [0.065, 0.385], *p* = 0.006), whereas no association was observed among Aβ‐negative individuals (*β* = −0.122, SE = 0.077, 95% CI [−0.276, 0.031], *p* = 0.117). Meanwhile, greater WASO was associated with higher tau burden among Aβ‐positive individuals (*β* = 0.302, SE = 0.090, 95% CI [0.124, 0.480], *p* = 0.001), but not among Aβ‐negative individuals (*β* = −0.082, SE = 0.072, 95% CI [−0.224, 0.061], *p* = 0.259).

### 3.6 Sensitivity analysis

To ensure that our findings were not driven by individuals with cognitive impairment who did not meet MCI criteria, we repeated all primary analyses after excluding individuals classified as “impaired not MCI” (*n* = 24) at the time of their tau‐PET scan. In these restricted models, higher WASO was significantly independently associated with greater tau‐PET burden (*β* = 0.138, *p *= 0.030). The interactions of *APOE* ε4 and amyloid status with TST were each non‐significant (*p* > 0.070). The interaction between TVPA and APOE ε4 remained non‐significant, and the interaction with amyloid status was attenuated and no longer reached significance after correction for multiple comparisons (*β* = 0.261, *p *= 0.021). In contrast, the moderating effects of WASO with both *APOE* ε4 carrier status (*β* = 0.377, *p *< 0.001) and amyloid positivity (*β* = 0.575, *p *< 0.001) remained robust. These results suggest that associations involving sleep continuity are relatively insensitive to the inclusion of participants with a diagnosis of “impaired not MCI,” whereas relationships involving physical activity may be more influenced by the inclusion of participants with a diagnosis of impaired not MCI.

In the second set of sensitivity analyses modeling global amyloid burden as a continuous variable, the interaction effect for TVPA for tau‐PET burden was no longer significant (*β* = 0.083, *p *= 0.157), and the interaction effect for WASO with tau‐PET burden remained robust (*β* = 0.182, *p *= 0.005). Modeling TST categorically (TST < 6 h [*n* = 4], 6 to 8 h [*n* = 72], >8 h [*n* = 44]) or using tertiles to explore non‐linear effects did not produce significant TST‐related findings (data not shown). In an additional sensitivity analysis, each lifestyle predictor was modeled separately without mutual adjustment for the other lifestyle variables. The overall pattern of results remained consistent with the primary analyses. Specifically, the interaction effects for WASO × Amyloid status, TVPA × Amyloid status, and WASO × APOE ε4 remained similar in direction and magnitude, and key findings remained statistically significant (*p* < 0.050). Finally, in sensitivity analyses restricting the sample to participants with actigraphy assessments within 750 days of tau‐PET imaging and using the temporally closest actigraphy visit (*n* = 94; Aβ‐positive = 29), the interaction effects between amyloid status and lifestyle measures remained significant. Specifically, the interactions for TVPA × Amyloid status (*p* = 0.010) and WASO × amyloid status (*p* = 0.040) were consistent in direction and statistical significance with the primary analyses (Figure S1).

## 4 DISCUSSION

This study examined whether associations between actigraphy‐derived physical activity and sleep measures and early tau‐PET burden differed as a function of genetic and amyloid status in cognitively unimpaired older adults. Consistent with our central hypothesis, we found that APOE ε4 carrier status and Aβ‐positive positivity moderated associations between sleep continuity, physical activity, and early tau pathology, highlighting risk‐dependent heterogeneity during the preclinical stage of AD.

Greater WASO was associated with higher tau‐PET burden among *APOE* ε4 carriers and among Aβ‐positive‐positive individuals, but not in lower‐risk groups. Additionally, TVPA was positively associated with early tau burden among Aβ‐positive‐positive individuals, whereas no association was observed among Aβ‐positive‐negative individuals. In contrast, no associations were observed in the full sample, underscoring the importance of risk stratification when evaluating lifestyle–pathology relationships in cognitively unimpaired individuals.

These findings extend prior work linking sleep disruption to AD pathology by suggesting that associations with tau may emerge primarily in individuals at elevated risk. A recent meta‐analysis of 30 studies spanning the AD spectrum reported that poorer sleep quality was associated with greater amyloid burden measured using CSF and Aβ‐positive, whereas associations with tau, assessed using CSF or tau‐PET, were not consistently observed.^16^ Importantly, the limited tau‐related findings may reflect methodological limitations, including small sample sizes (77 participants across two studies), sole reliance on self‐reported measures, and the use of tau tracers with reduced sensitivity to early‐stage tau burden.

Our findings are also partly consistent with a small, but growing, body of tau‐PET research suggesting that associations between sleep and tau pathology may depend on underlying AD risk. For example, a study of 46 older women without dementia reported that shorter sleep duration was associated with greater Braak I‐II tau‐PET burden, but only among *APOE* ε4 carriers.^19^ The present study extends prior work by focusing exclusively on cognitively unimpaired individuals, including both men and women, and by jointly examining objective measures of sleep continuity and duration, alongside physical activity. Additional evidence supporting a link between sleep disruption and tau pathology comes from studies using complementary biomarkers and methodologies. Shorter actigraphy‐estimated TST is associated with greater CSF‐measured phosphorylated and total tau burden among cognitively unimpaired older adults, with stronger associations reported among *APOE* ε4 carriers.^52^ *Post mortem* studies similarly report greater tau tangle burden in relation to poorer actigraphy‐measured sleep quality, particularly among *APOE* ε4 carriers.^5^ In parallel, polysomnography‐based research in cognitively unimpaired older adults has shown that less slow‐wave sleep is associated with greater tau‐PET signal in medial and inferior temporal regions.^53^ Although these studies did not explicitly test moderation by amyloid burden, taken together they support the hypothesis that objective sleep disruption is linked to greater tau pathology among individuals at elevated risk for AD and suggest that sleep continuity may be a sensitive behavioral correlate of early tau accumulation during the preclinical stage.

In contrast, higher physical activity was unexpectedly associated with greater tau‐PET burden among Aβ‐positive individuals. Importantly, this association was observed in participants who were cognitively unimpaired at the time of tau‐PET imaging, indicating the presence of amyloid burden in the absence of overt clinical symptoms. Although counterintuitive, this positive association between physical activity and early tau‐PET burden could be consistent with cognitive resilience frameworks, which posit that modifiable lifestyle factors, such as physical activity, may buffer the clinical expression of AD pathology rather than prevent or alter its accumulation.^32^ Supporting this interpretation, neuropathological data have shown that greater late‐life physical activity was associated with higher levels of *post mortem* tau pathology, yet physical activity attenuated the negative impact of pathology on cognition.^54^ Mechanistically, *post mortem* studies have shown that greater physical activity is associated with better synaptic integrity and cognitive performance, despite substantial AD pathology.^55^ An alternative, but not mutually exclusive, explanation is the presence of a selection bias within cognitively unimpaired samples, whereby individuals with lower activity and greater vulnerability may have already transitioned to impairment. As a result, Aβ‐positive individuals who remain cognitively unimpaired may represent a relatively resilient subgroup, potentially contributing to the observed association between higher physical activity and greater tau burden.

Recently, Yau et al. provided the first evidence linking physical activity to longitudinal tau‐PET accumulation, reporting that higher step counts predicted slower tau accumulation and reduced cognitive decline among cognitively unimpaired at baseline, Aβ‐positive individuals.^8^ Although these findings differ directionally from the cross‐sectional associations observed in the present study, the results may not be incompatible. One possibility is that physical activity exerts resilience‐promoting effects,^32^ such that individuals with greater activity levels are better able to maintain cognitive function despite ongoing tau accumulation, particularly once substantial amyloid pathology is present. Alternatively, the observed differences may reflect methodological factors, including the relatively small Aβ‐positive subgroup, longitudinal versus cross‐sectional study design, inclusion of individuals who progressed to MCI in the Yau et al. cohort, and differences in the timing of activity assessment relative to tau‐PET acquisition. Measurement differences may also be relevant, as step counts primarily index ambulatory activity, whereas actigraphy‐derived total volume of physical activity captures a broader range of movement behaviors. Additional differences in tracer selection (^1^ ^8^F‐MK6240 vs ^1^ ^8^F‐flortaucipir) and sample composition may further contribute to the divergent findings. Collectively, these considerations underscore the importance of disease stage, timing, and measurement modality in shaping observed associations between physical activity and tau pathology and highlight the need for longitudinal studies with extended follow‐up to clarify the temporal dynamics linking physical activity, tau accumulation, and cognitive resilience.

Taken together, these findings suggest that associations of sleep and physical activity with early tau burden differ according to level of AD risk, including underlying amyloid burden and genetic risk. To evaluate the robustness of these interpretations, we conducted a series of sensitivity analyses. Results indicated that associations involving sleep quality were robust across analytic specifications, including after exclusion of individuals with subthreshold cognitive impairment and alternative modeling of amyloid burden. In contrast, the interaction between physical activity and amyloid status was attenuated and no longer statistically significant after exclusion of participants classified as “impaired not MCI” and when amyloid burden was modeled as a continuous variable. This may reflect the relatively high proportion of Aβ‐positive individuals among those classified as impaired not MCI (*n* = 10 [43%]). These findings could also suggest that wakefulness after initial sleep onset may represent a more stable behavioral correlate of early tau burden among at‐risk individuals, whereas associations involving physical activity may be more contingent on disease stage, early cognitive changes, and analytic specification.

While this study provides insights into the associations between actigraphy‐based measures of physical activity and sleep and early tau‐PET burden, it has several limitations. The cross‐sectional design and substantial temporal gap between actigraphy and tau‐PET (mean ∼2.6 years, with considerable variability) limit inference regarding causality and temporal ordering. Reverse causation is a plausible explanation, as early AD‐related neurobiological changes may influence sleep continuity and physical activity patterns prior to or concurrent with detectable tau accumulation, rather than lifestyle factors driving tau deposition. In addition, temporal misalignment between lifestyle and imaging measures may reduce the extent to which actigraphy reflects lifestyle at the time of tau‐PET. Although models were adjusted for time between assessments, statistical adjustment cannot fully address this limitation. Notably, sensitivity analyses restricting the sample to more temporally proximal assessments yielded consistent interaction effects, suggesting that findings are not solely driven by extended time intervals.

Subgroup sample sizes were modest, particularly among Aβ‐positive individuals and APOE ε4 carriers, limiting statistical power and supporting interpretation of interaction effects as exploratory. In addition, because analyses were restricted to cognitively unimpaired individuals, it is possible that individuals with lower physical activity, poorer sleep, and greater underlying pathology had already transitioned to impaired diagnostic groups and were therefore not included, which may influence observed associations. Although we focused on TST and WASO as primary indices of sleep duration and continuity, other sleep characteristics (e.g., sleep efficiency, variability, and timing) may capture additional dimensions of sleep physiology that are not fully represented by these measures and may provide complementary insight into associations with AD pathology. Finally, the sample consisted of highly educated, predominantly white, research‐engaged participants from a longitudinal cohort enriched for AD risk, which may limit generalizability to broader and more diverse populations.

In summary, our findings highlight the importance of accounting for individual differences in AD genetic risk and amyloid status when evaluating associations of lifestyle factors with biomarkers in cognitively unimpaired individuals. Objectively measured sleep and physical activity may relate differently to tau burden depending on an individual's underlying risk profile and cognitive status. Specifically, poor sleep continuity may reflect early vulnerability to tau burden in APOE ε4 carriers and Aβ‐positive‐positive individuals, whereas higher physical activity may indicate a compensatory or resilience‐promoting response in those with amyloid pathology. Collectively, these findings highlight the importance of considering individual differences in genetic and amyloid status when examining lifestyle contributions to early AD pathophysiology and suggest potential pathways for personalized prevention strategies.

## AUTHOR CONTRIBUTIONS

DDC conceived and led the analytic plan, processed the actigraphy data, conducted statistical analyses, interpreted the data, and drafted the initial version of the manuscript. NR led the tau‐PET data processing, regional tau quantification, and interpretation of neuroimaging findings. JCS contributed to the interpretation of physical activity measures and manuscript review. AS and CP contributed to study design, interpretation of biomarker findings, and revision of the manuscript. APS contributed to the implementation of actigraphy data, conceptualization and interpretation of sleep measures, and review of the manuscript. MA and AB contributed to study oversight, interpretation of findings within the context of the BIOCARD study, and revision of the manuscript. The BIOCARD Research Team contributed to participant recruitment, data acquisition, and maintenance of the longitudinal cohort. All authors reviewed and approved the final manuscript.

## CONFLICT OF INTEREST STATEMENT

DDC, NR, JCS, AS, CP, and MA have nothing to disclose. APS has served as a paid consultant to Sequoia Neurovitality, BellSant, Inc., Amissa, Inc., and Synaptic Health, LLC. AB is an inventor on JHU intellectual property with patents pending and licensed to AgeneBio, Inc. AB is a consultant to AgeneBio, Inc. and was a consultant to Lantheus Medical Imaging, Inc. These arrangements have been reviewed and approved by the JHU in accordance with its conflict‐of‐interest policies. Author disclosures are available in the Supporting Information.

## CONSENT STATEMENT

All human subjects provided informed consent.

## Supporting information




**Supporting Information**: alz71555‐sup‐0001‐ICMJE.pdf

**Supporting Information**: alz71555‐sup‐0002‐SuppMat.docx

## Data Availability

Data used in these analyses are available through standard application procedures described on the BIOCARD website (www.biocard‐se.org). Additional information or materials relating to the analysis are available from the corresponding author (DDC) upon reasonable request.
