# Supplementary material for: APOE ε4 and amyloid status moderate the associations between sleep, physical activity, and tau‐PET burden in cognitively unimpaired older adults
Source: Alzheimers Dement. 2026 Jun 16;22(6):e71555. doi: 10.1002/alz.71555 (PMC13272105; doi:10.1002/alz.71555)
Supplement: Supplementary file 2 — Supporting Information: alz71555‐sup‐0002‐SuppMat.docx [file ALZ-22-e71555-s002.docx]

**Supplementary Files**

**Supplementary Table 1.** Participant characteristics by Aβ-PET status

| **Participant characteristic** | **Full sample**  **(n = 120)** | ***Aβ-PET*–negative**  **(n = 87)** | ***Aβ-PET*–positive**  **(n = 33)** | ***p*-value** |
| --- | --- | --- | --- | --- |
| Female sex, n (%) | 71 (59%) | 52 (61%) | 19 (59%) | 0.900 |
| Age | 69.5 ± 9.3 | 68.1 ± 10.0 | 73.2 ± 5.7 | <0.001 |
| Education, years | 17.5 ± 2.0 | 17.4 ± 2.0 | 17.7 ± 1.8 | 0.400 |
| *APOE ε4* carrier, n (%) | 43 (36%) | 25 (29%) | 18 (55%) | 0.016 |
| TVPA^a^ | 2,859 ± 325 | 2,875 ± 277 | 2,818 ± 430 | 0.454 |
| Total sleep time (min) | 459.4 ± 55 | 460.8 ± 54 | 455.7 ± 58 | 0.652 |
| Wake after sleep onset (min) | 40.8 ± 21 | 40.1 ± 19 | 42.6 ± 24 | 0.605 |
| Tau-PET SUVR in Braak I and II^b^ | 1.9 ± 0.7 | 1.7 ± 0.4 | 2.4 ± 0.9 | <0.001 |
| Time from actigraphy to tau PET (days) | -958 ± 1,121 | -962 ± 1,120 | -948 ± 1,141 | 0.946 |
| Vascular Risk Score | 1.2 ± 0.9 | 1.2 ± 0.9 | 1.2 ± 0.9 | 0.720 |
| *Note:* Values are mean ± standard deviation unless otherwise indicated.  Abbreviations: PET = positron emission tomography; SD = standard deviation; SUVR = standardized uptake value ratio; TVPA = total volume of physical activity.  ᵃTotal volume of physical activity represents the average log-transformed total activity epoch counts from the 10 most active hours per day.  ᵇTau-PET SUVR values reflect the mean uptake across Braak stages I and II. | | | | |

**Supplementary Table 2.** Participant characteristics by APOE ε4 carrier status

| **Participant characteristic** | **Full sample**  **(n = 120)** | ***APOE ε4 non-carrier* (n = 87)** | ***APOE ε4 carrier***  **(n = 33)** | ***p*-value** |
| --- | --- | --- | --- | --- |
| Female sex, n (%) | 71 (59%) | 50 (65%) | 21 (49%) | 0.127 |
| Age | 69.5 ± 9.3 | 70.5 ± 9.1 | 67.7 ± 9.6 | 0.126 |
| Education, years | 17.5 ± 2.0 | 17.3 ± 2.0 | 17.9 ± 1.8 | 0.101 |
| *Aβ-PET status*, n (%) | 33 (28%) | 15 (19%) | 18 (42%) | 0.016 |
| TVPA^a^ | 2,859 ± 325 | 2,858 ± 303 | 2,861 ± 365 | 0.968 |
| Total sleep time (min) | 459.4 ± 55 | 457.7 ± 53 | 462.5 ± 60 | 0.663 |
| Wake after sleep onset (min) | 40.8 ± 21 | 39.4 ± 19 | 43.2 ± 23 | 0.372 |
| Tau-PET SUVR in Braak I and II^b^ | 1.9 ± 0.7 | 1.8 ± 0.5 | 2.0 ± 0.9 | 0.071 |
| Time from actigraphy to tau PET (days) | -957.9 ± 1,121 | -1,139.4 ± 1,087 | -633.0 ± 1,121 | 0.019 |
| Vascular Risk Score | 1.18 ± 0.9 | 1.26 ± 1.0 | 1.05 ± 0.8 | 0.200 |
| *Note:* Values are mean ± standard deviation unless otherwise indicated.  Abbreviations: PET = positron emission tomography; SD = standard deviation; SUVR = standardized uptake value ratio; TVPA = total volume of physical activity.  ᵃTotal volume of physical activity represents the average log-transformed total activity epoch counts from the 10 most active hours per day.  ᵇTau-PET SUVR values reflect the mean uptake across Braak stages I and II. | | | | |


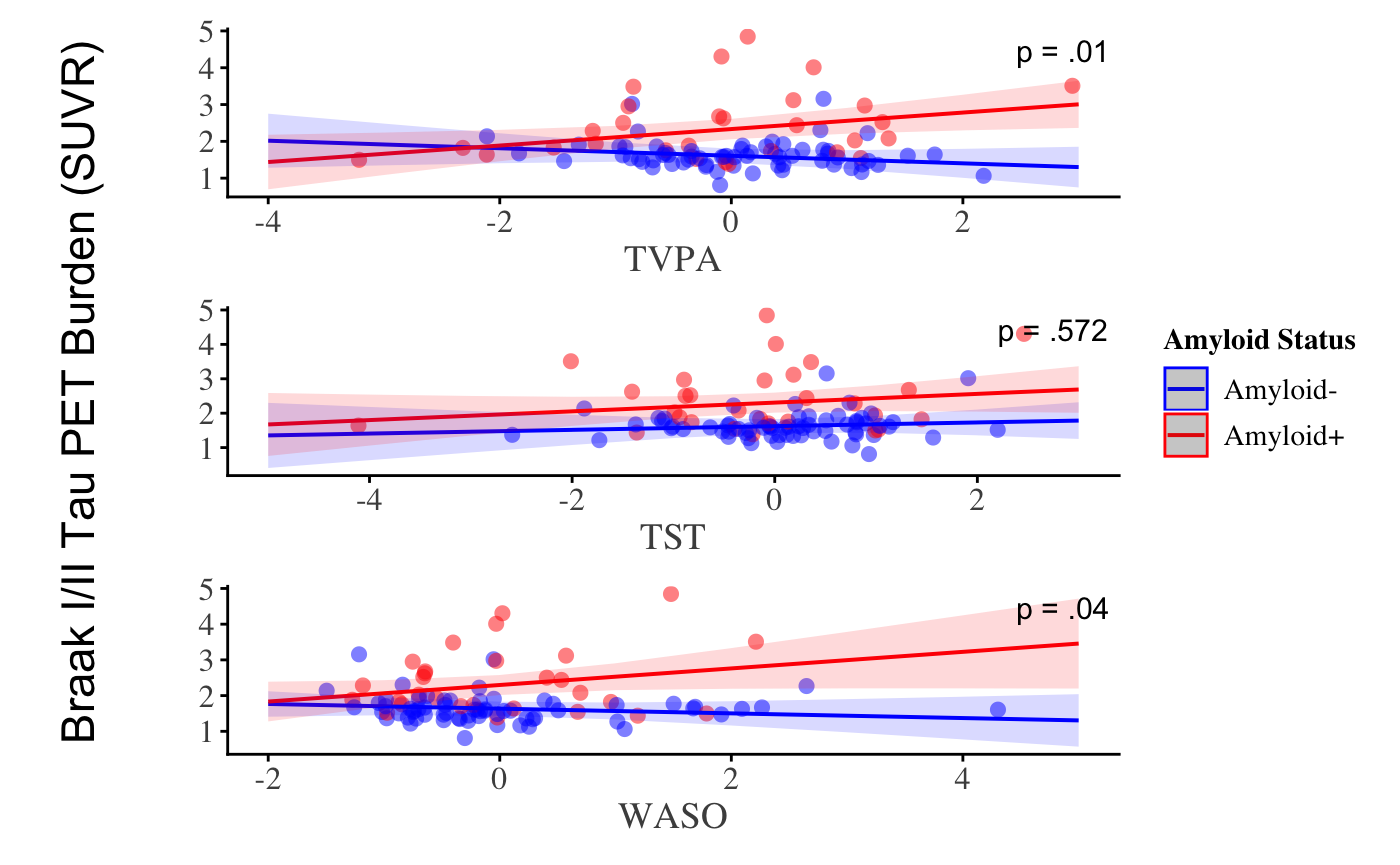


**Supplementary Figure 1:** Sensitivity analysis examining the moderating effect of amyloid-PET positivity on the associations between total volume of physical activity (TVPA), total sleep time (TST), and wake after sleep onset (WASO) and early tau-PET burden. Analyses were restricted to participants with actigraphy assessments within 750 days of tau-PET imaging and used the temporally closest actigraphy visit (n = 94; amyloid-positive = 29).
